# Biopsychosocial approach in cardiological care: a scoping review of integrated treatments for personalized patient care

**DOI:** 10.3389/fcvm.2026.1727783

**Published:** 2026-03-05

**Authors:** Dina Di Giacomo, Enrica Cogodi, Lucrezia Piermarini, Antonio Scarà, Claudio Ferri, Luigi Sciarra, Silvio Romano

**Affiliations:** 1 Life, Health and Environmental Sciences Department, University of L’Aquila, L’Aquila, Italy; 2 Post-Graduate School in Clinical Psychology, University of L’Aquila, L’Aquila, Italy; 3 Di Lorenzo Clinic, Avezzano, L’Aquila, Italy; 4 Internal Medicine Division, ASL1 Abruzzo, L’Aquila, Italy; 5 Heart Failure Clinic, ASL 1 Abruzzo, L’Aquila, Italy

**Keywords:** biomarkers, biopsychosocial approach, cardiovascular disease, cardiovascular risks, psychological evaluation

## Abstract

**Objective:**

We conducted a scoping review of the literature focusing on combining biomarker detection and psychological evaluation according to the biopsychosocial approach to expound the multifactorial aspects of cardiological care.

**Method:**

The search strategy was restricted to the 2014–2024 period. The core of this evidence-based analysis was the investigation of emotional and behavioural signs in cardiovascular (CV) risk/disease, the characteristics of adherence, and the efficacy of psychological treatments, especially highlighting the evidence of studies based on pre–post study design models for the investigation of psychological treatment efficacy in CV risk/disease.

**Results:**

The literature includes systematic reviews and meta-analyses on patient education for the effectiveness of psychological interventions on psychosocial aspects and, consequently, on adherence to therapeutic prescriptions. From this perspective, it is important to adopt the biopsychosocial approach in the prevention, evaluation, and management of CV disease to understand all determinants of disease and multidisciplinary treatments. Topics are arranged into six themes: (1) epidemiological aspects of the included studies, (2) medical and psychological insights into CV research protocols from an integrated biopsychological perspective, (3) evidence of emotional and behavioural symptoms in CV risk, (4) evidence of emotional and behavioural symptoms in CV disease, (5) characteristics of adherence and psychological treatment, and (6) evidence of pre- and post-psychological treatment in CV risk/disease.

**Conclusions:**

This review demonstrates that interventions are required to address patient factors, such as emotional, behavioural, and cognitive dimensions, that can improve the quality of life and adherence to medication. The link between physical and mental care is crucial and should be supported by extensive evidence-based outcomes in terms of tailored actions in health monitoring and management of CV risk/disease clinical targets.

## 1 Introduction

Cardiovascular disease (CVD) is the leading cause of death in all age groups worldwide and also the leading cause of hospital discharges. Lifestyle habits and modifiable physiological and biochemical factors are significant clinical features. The diagnostic process is based on clinical signs and symptoms, laboratory data, and patient reports. In recent clinical practice guidelines, the European Society of Cardiology (ESC) highlighted that social determinant components of patient care and understanding the ability of patient communication regarding one's own symptoms represent a significant integration of medical settings for better care (1).

The multifactorial perspective in cardiological care focuses on the diagnosis, treatment, and management of heart and blood vessel conditions in patients with chronic diseases. Individualized lifestyle medicine therapies aim to optimize the effects of pharmacological therapies and improve patient adherence to a positive quality of life (QoL) (2–4). Cardiological medication therapies manage heart conditions by targeting key factors such as blood pressure (BP), cholesterol, and clotting, using classes such as angiotensin converting enzyme (ACE) inhibitors/angiotensin receptor blockers (ARBs), beta-blockers, calcium channel blockers, diuretics, anticoagulants/antiplatelets, and statins, to reduce the heart's workload, improve blood flow, prevent clots, and manage rhythm, with newer options such as SGLT2 inhibitors (or “gliflozins”) and angiotensin receptor-neprilysin inhibitors (ARNIs) evolving treatment to improve outcomes for conditions such as heart failure (HF), often tailored through personalized medicine (5). A multidimensional framework to tailor the integrated approach according to the patient is the biopsychosocial model (BPS), focused on the implementation of QoL in patient care: joining mental and physical treatments, CV patients may improve their adherence and related health outcomes (6–9). Tailored awareness, well- and progressively defined goals (step by step), and fitted emotion health feelings could be key points in the medical–patient relationship to reinforce and improve QoL and persistent behaviours—basic requirements for adherence to medications—and boost coping strategies for effective compliance (10). According to the BPS approach, the most relevant psychosocial risk factors for CV disease are work-related stress and/or stress related to family life, lack of social support or social isolation, depression, anxiety, anger-hostility, and a specific personality trait (Type D Personality). Psychosocial factors contribute to the increased risk of CV disease through an unhealthy lifestyle (smoking, unhealthy food, and reduced exercise), increased use of healthcare, and poor medical and behavioural adherence (11). Low behavioural adherence was associated with smoking habits and higher levels of perceived stress (12). The main reasons for non-adherence, especially to statins for the treatment of hypercholesterolaemia, include polypharmacological susceptibility to drug side effects, cognitive dysfunction, physical disability, and depression (13).

In the literature, there are both systematic reviews and meta-analyses on patient education for the effectiveness of psychological intervention on psychosocial aspects and, consequently, on adherence to therapeutic prescriptions (8).

Despite the relevance of the BPS approach, the application of this model in research protocols remains under-explored. Therefore, we performed a review of the features of the BPS approach for CV conditions. Specifically, the research objectives of this review are as follows:

a. summarize the current evidence for investigations connecting the BPS approach and CV clinical paths for health management,
b. identify gaps in the literature that may require further research, and
c. draw implications for further research and clinical practice.

## 2 Materials and methods

### 2.1 Study design

We conducted a review to analyze the literature focusing on the incidence of biomarker detection and psychological evaluation according to the BPS approach. We followed the scoping review framework outlined by Arksey and O’Malley (14). This is the most commonly used framework for scoping reviews. In line with the recommended guidance and common practice (15), we did not assess the methodological quality of included studies, as the aim of the scoping review was to identify whether there is evidence, rather than to determine the quality of the evidence base. We performed this review according to the Preferred Reporting Items for Systematic Reviews and Meta-analysis (PRISMA)—Extension for Scoping Reviews (PRISMA-R) (16).

### 2.2 Search strategy

To identify potentially relevant studies for inclusion, we performed a search of MEDLINE through PubMed, Scopus, Web of Science, Scopus, and PsycInfo platforms in January 2025. The key search terms were “cardiovascular disease”, “biopsychological approach”, “psychological aspects”, “psychological dynamics”, “adherence”, “polypharmacology”, “compliance”, “cardiological care”, “health management”, “peripheral artery disease”, and “chronic kidney disease” in various combinations.

### 2.3 Inclusion and exclusion criteria

To be considered eligible, the titles and abstracts of the retrieved studies had to refer to patients with CV and psychological and clinical evaluations. The search strategy was restricted to the period 2014–2024. To be included, studies had to (a) be published in English, (b) include biomarkers, (c) include psychological measures, and (d) include the BPS approach. In addition, we excluded all reviews on the topic.

### 2.4 Article selection and data extraction

To ensure the reliability of the review, the titles and abstracts of the retrieved studies were independently screened by two raters for eligibility. The same two raters also evaluated the full texts for inclusion that were retrieved from online databases and faculty interlibrary services. Any disagreement between the raters was resolved through discussion until a consensus was reached. Following data extraction, the data were synthesized and discussed by the research team. After summarizing the study characteristics, to provide an overview of the studies included, the data were tabulated using Microsoft Excel® and characterized using simple descriptive statistics. All data were presented as absolute and relative frequencies. The summarized data are presented as tables and figures. Ethical approval was not required for this review of the existing literature.

The following data were extracted from each paper: (a) general information: authors, publication year, country, and involved institution (e.g., University and Medical Centre); (b) study characteristics: study design, chronic disease, and range age; (c) biomarkers and psychological measures used; and (d) data related to the research question of the review: incidence of biomarker detection and psychological evaluation according to a BPS approach, and a summary of outcomes.

### 2.5 Data synthesis

General information, study characteristics, psychological measures used, and data related to the research question of the review were descriptively synthesized. To explore the presence of clinical data and psychological measures, the results were summarized referring to the BPS approach outcomes.

### 2.6 Selected studies

A literature search of the PubMed and Web of Science electronic databases provided a total of 1,392 publications. After removing 399 duplicates, 993 references were identified for screening. Based on the inclusion/exclusion criteria, two reviewers screened all titles and abstracts for eligibility and successfully resolved disagreements by consensus. First, 936 papers were excluded. The full texts of the remaining 301 papers potentially worthy of inclusion were comprehensively examined.

**Figure 1:**
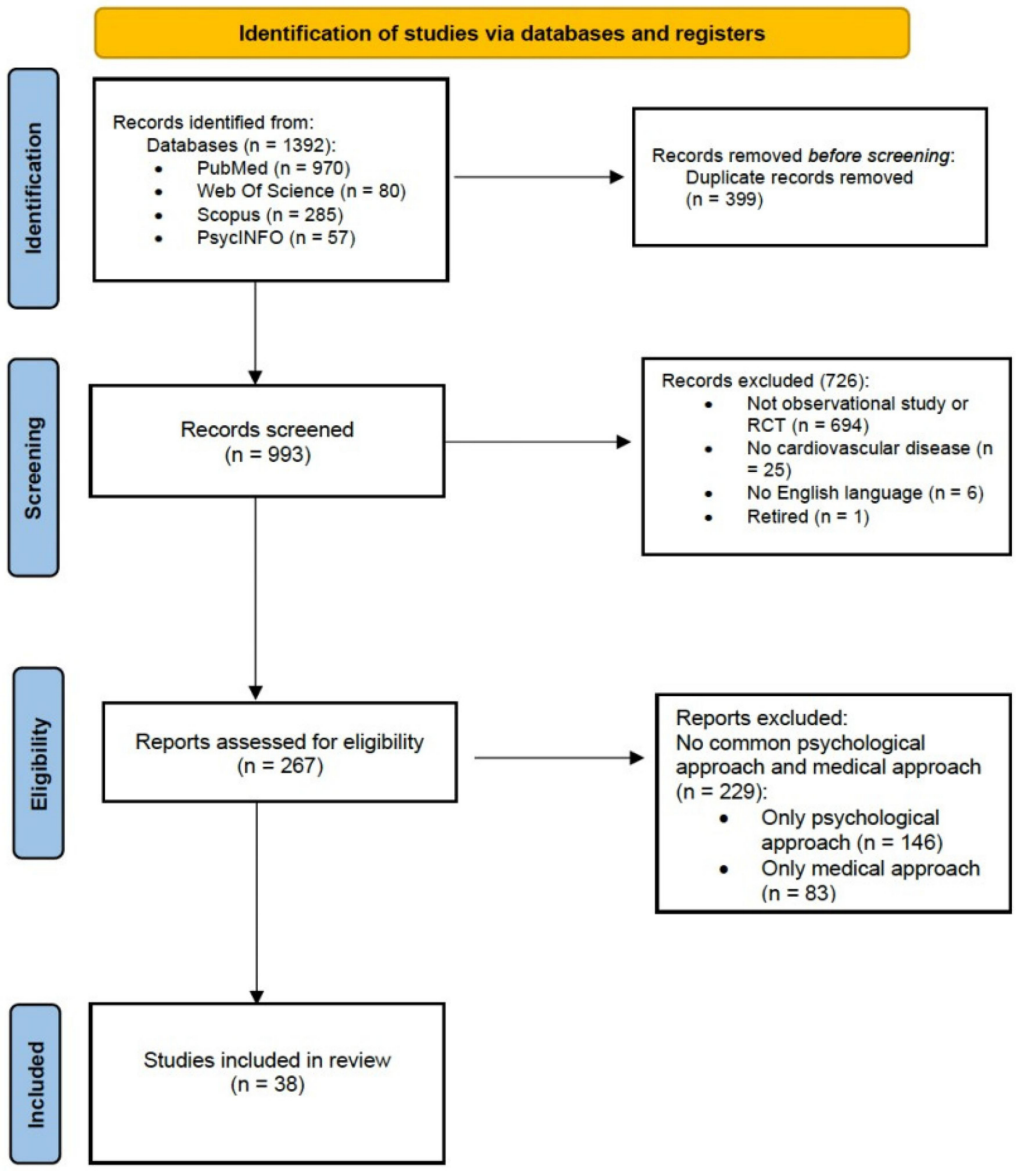
A PRISMA flowchart of the study selection process for the review.

Of the total of 301 articles assessed for eligibility, 263 were excluded. The reasons for the first and second exclusions were also reported. Finally, 38 studies were included. Figure 1 illustrates the search and selection process.

## 3 Results

### 3.1 Characteristics of included studies

The main characteristics of the included studies are summarized in Supplementary Tables S1a,b. We identified the following from each included study: (a) study design, (b) authors, (c) CV condition, (d) biomarkers or clinical data, (e) medical adherence, (f) behavioural adherence, (g) psychological measures used, (h) psychological treatment, and (i) outcomes.

**Figure 2:**
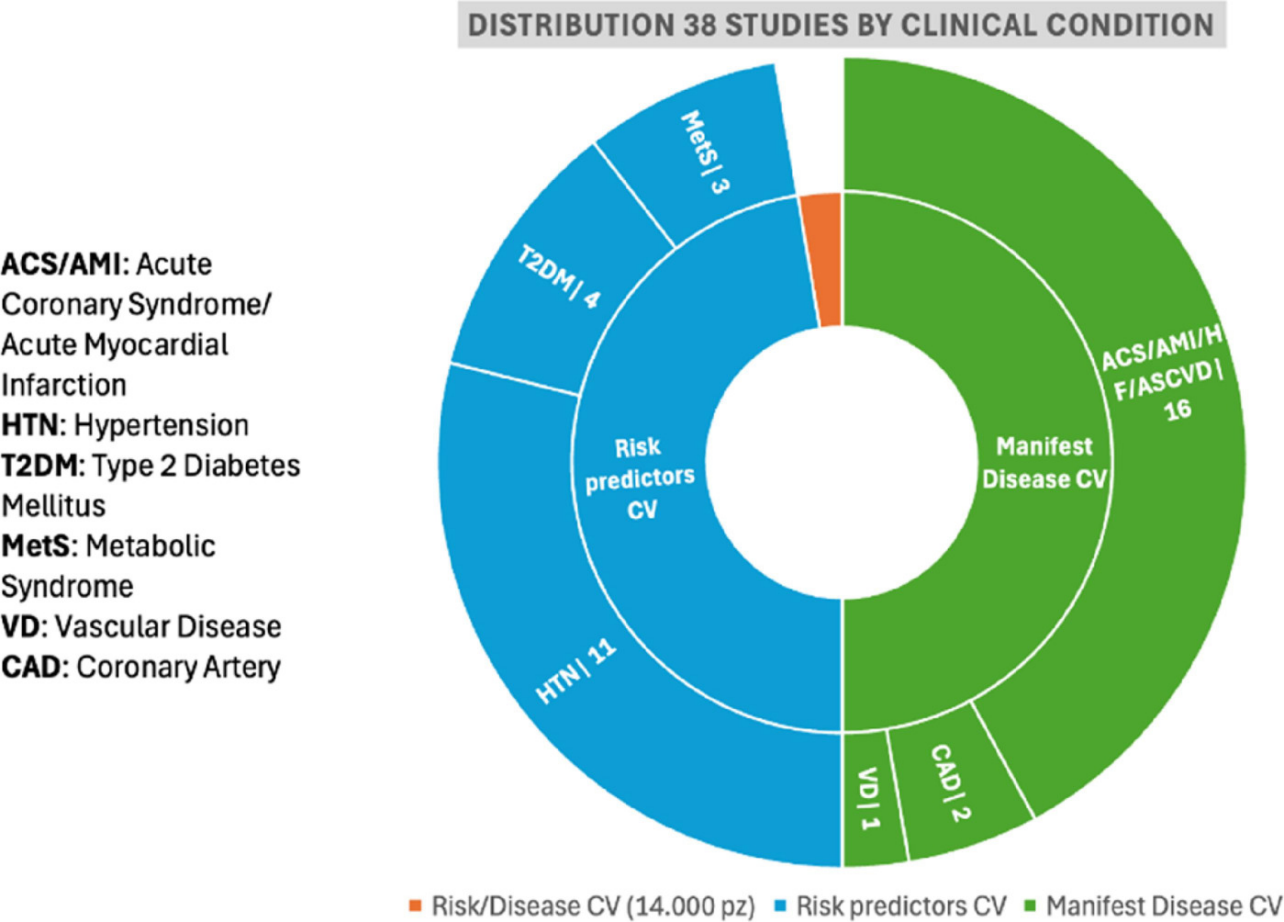
Representation of data distribution in the CV path.

This study was divided into CV risk factors (n = 19; Supplementary Table S1a) and CV disease (n = 18; Supplementary Table S1b). One study was based on a comprehensive sample of both CV risk factors and disease. Figure 2 presents the distribution data.

As reported, the selected studies for CV disease were developed considering acute coronary syndromes (ACS), acute myocardial infarction (AMI), vascular disease (VD), and coronary artery disease (CAD), also known as ischaemic heart disease (IHD), HF, and atherosclerotic cardiovascular disease (ASCVD). Studies have investigated HF, including chronic heart failure (CHF) and heart failure with reduced ejection fraction (HfrEF). CAD or IHD included ischaemia without obstructive coronary arteries (INOCA). Studies dedicated to CV risk factors included hypertension (HTN), type 2 diabetes mellitus (T2DM), and metabolic syndrome (MetS). HTN included resistant HT (RHTN). MetS was defined according to the National Cholesterol Education Program (17) and the Consensus of both the International Diabetes Federation (IDF) and the American Heart Association/National Heart, Lung, and Blood Institute (AHA/NHLBI) (18).

Analyzing the detected studies regarding peripheral arterial disease (PAD) and nephropathy following diabetes and/or HTN, also known as cardiorenal nephropathy, it is evident that they applied a significantly medical approach, based on biological data, biomarkers, and/or clinical data, neglecting the patient factor. In fact, none of these studies investigated the psychological dimensions of the integrated patient approach.

The 38 included studies were examined from different perspectives, analyzing the features of research efforts applying the BPS approach. First, we processed the studies from an epidemiological perspective, drawing an international scientific scenario. Then, we examined the medical and psychological insights of CV research protocols by applying the BPS framework. The core of the evidence-based analysis was the investigation of emotional and behavioural signs in CV risk/disease, the characteristics of adherence, and the efficacy of psychological treatments, particularly highlighting the evidence of studies based on pre–post study design models for the investigation of psychological treatment efficacy in CV risk/disease. The topics were arranged into six themes:

*Theme 1:* Epidemiological aspects of the included studies
*Theme 2:* Medical and psychological insights into CV research protocols from an integrated biopsychological perspective
*Theme 3:* Evidence of emotional and behavioural symptoms in CV risk
*Theme 4:* Evidence of emotional and behavioural symptoms in CV disease
*Theme 5:* Characteristics of adherence and psychological treatment
*Theme 6:* Evidence of pre- and post-psychological treatment in CV risk/disease

## 4 Discussion

### 4.1 Theme 1: epidemiological aspects of the included studies

**Figure 3:**
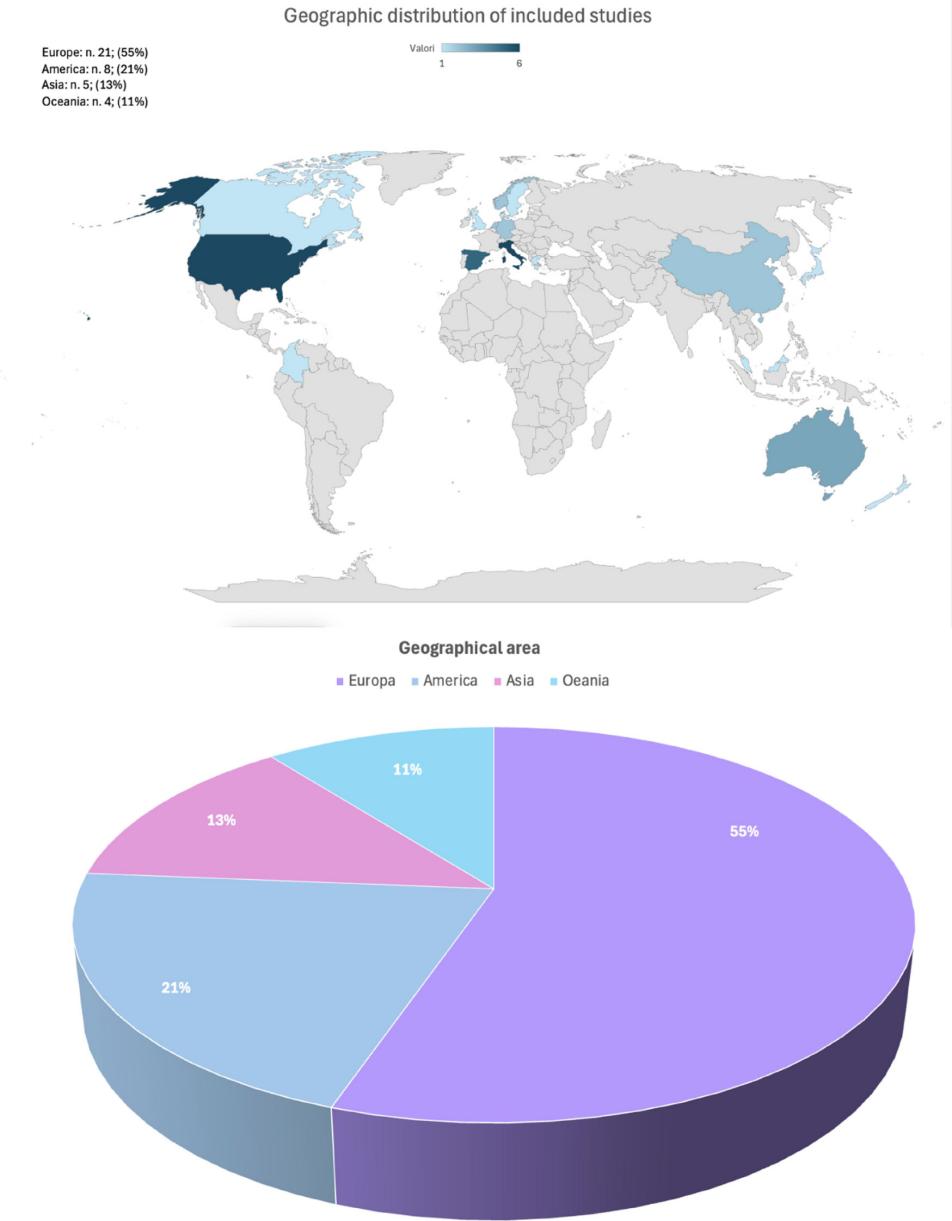
Distribution of CV studies by country and geographical macro-area of the first author.

First, we analyzed the epidemiological aspects of the extracted research based on geographical incidence, as shown in Figure 3: CV research programmes are more active in Europe (55%) than in America (21%), Asia (13%), and Oceania (11%). Then, analyzing the country efforts, the scientific interest in CVD seemed equally prevalent in the USA (16%) and Italy (16%), followed by Spain (13%). With regard to research interest over time, we analyzed the frequency of studies in the 2014–2024 period, evidencing the growth in interest by increasing research; we observed a reduction in publications in the 2022–2023 period that could be attributed to the impact of the COVID-19 pandemic.

The incidence of active participation of psychologists in the cardiological research protocol was analyzed based on authorship. It appeared to be increasing in the last 5 years, rendering mental health more relevant to cardiological care research protocols (Figure 3).

**Figure 4:**
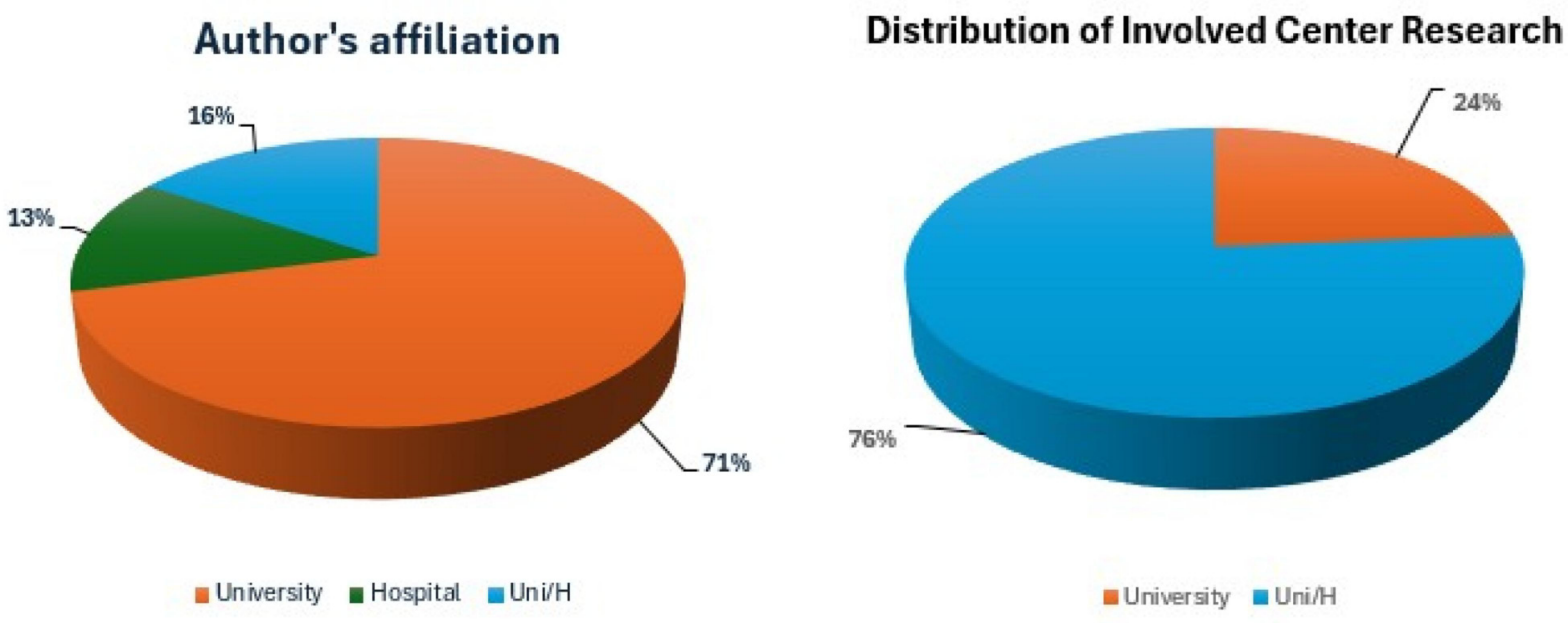
Author's affiliation and involved research centres.

Finally, we analyzed institutional involvement by author affiliations: academic researchers appeared more active (71%) than hospital/research centres; furthermore, studies based on the hospital/university partnership amounted to 16% (Figure 4).

### 4.2 Theme 2: medical and psychological insights into CV research protocols from an integrated biopsychological perspective

Our primary interest was to analyze the medical and psychological characteristics of the examined studies on CV targets from the BPS integrated perspective. With regard to the medical aspects, we examined the clinical features of the involved population in terms of disease, biomarkers, and laboratory data. Based on psychological insight, we analyzed the emotional, behavioural, and cognitive implications of CV conditions.

The medical measures for the involved population are biomarkers and/or clinical data. The main biomarkers or clinical data studied were as follows: body mass index (BMI; *n* = 32, 84.2%), blood pressure (BP; *n* = 29, 76.3%), total cholesterol (TC; *n* = 12, 31.6%); low-density lipoprotein-cholesterol (LDL-C; *n* = 9, 23.7%), high-density lipoprotein-cholesterol (HDL-C; *n* = 10, 26.3%), triglycerides (TG; *n* = 7, 18.4%), waist circumference (WC; *n* = 7, 18.4%), and glycosylated haemoglobin (HbA1c; *n* = 9, 23.7%). With regard to the studies on CV risk factors, the Framingham Cardiovascular Risk Score was used (*n* = 1, 2.6%). In the studies that focused on CV disease, left ventricular ejection fraction (LVEF; *n* = 6, 15.8%) and heart rate (HR; *n* = 4, 10.5%) were measured.

Analyzing the overall data, it was found that CV risk was more relevant for investigating the disease impact on the cognitive functioning of CV patients, neglecting the relevance of emotional dimension on health management; in contrast, in CV disease, it seemed that the emotional dimensions were analyzed more, missing out the significant implications of integrating the functioning of emotion and cognition into health management by active patients.

**Figure 5:**
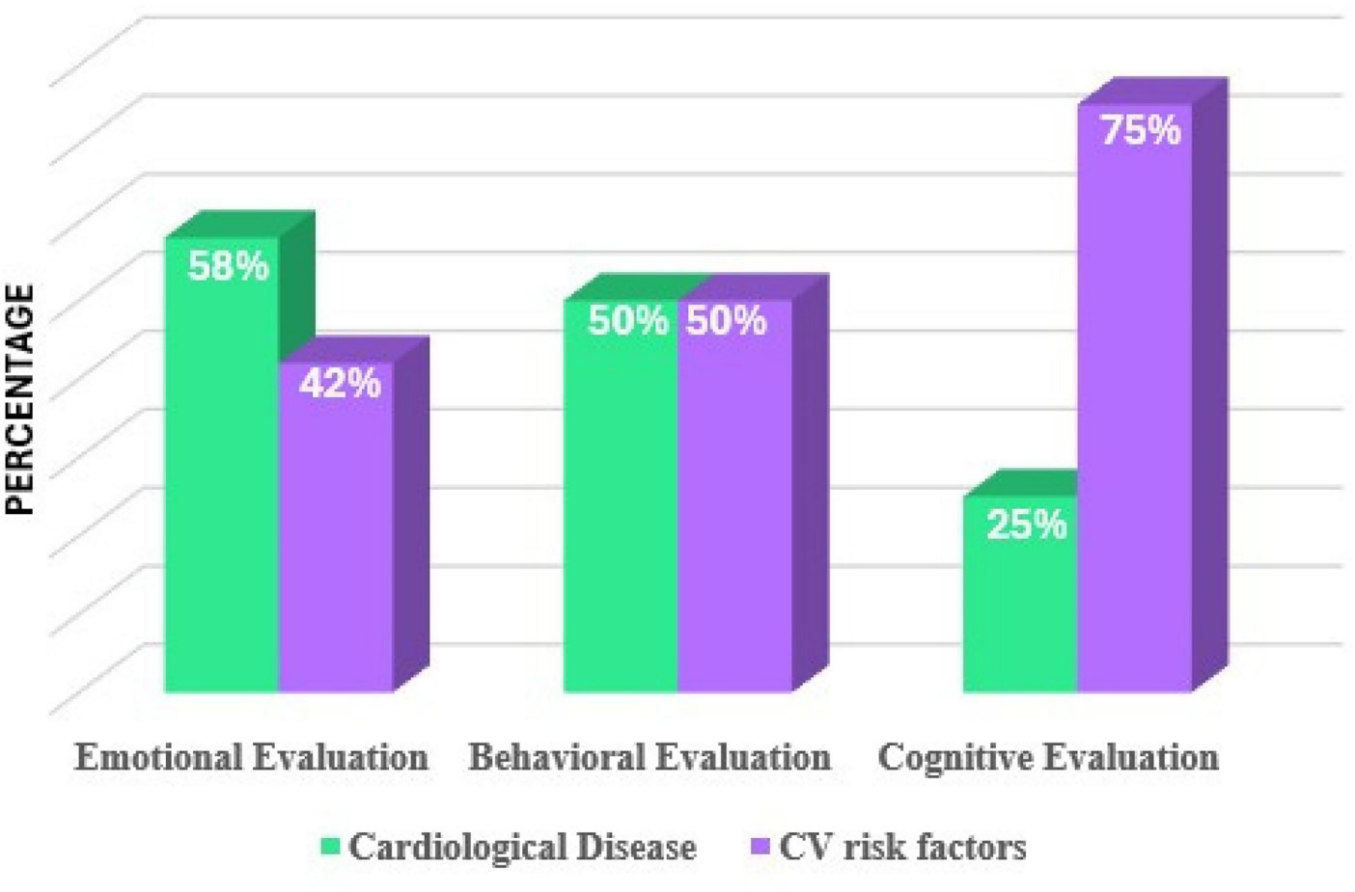
Psychological measures used in the studies.

Considering the psychological assessments, the relevant investigated psychological area was the cognitive area in CV risk factors, while the significant related area was the emotional area in CV disease. The cognitive tests were related to cognitive decline, attention, and memory (*n* = 8; 21%). Considering the emotional dimensions of CV disease, the most commonly applied tests were related to anxiety and depression traits. The emotional aspect was mainly investigated using the Hospital Anxiety and Depression Scale (HADS; *n* = 9, 23.7%) and Patient Health Questionnaire (PHQ; *n* = 7, 18.4%). Behavioural testing was based largely on QoL and self-care assessment. The most applied behavioural tests were the Short Form Health Survey (SF; *n* = 10, 26.3%) and EuroQoL-5 Dimension (EQ-5D; *n* = 5, 13.2%). The Type D Personality Assessment (DS-14; *n* = 2, 5.3%) was used for the diagnosis of Type D personality, the Post-Traumatic Diagnostic Scale (PTDS; *n* = 1, 2.6%) was used for the diagnosis of post-traumatic stress disorder, and the State Trait Anxiety Inventory (STAI; *n* = 2, 5.3%) was used for the assessment of state and trait anxiety. The Structured Clinical Interview for DSM-IV-TR Axis I Disorders (SCID; *n* = 1, 2.6%) and Mini International Neuropsychiatric Interview (MINI; *n* = 4, 10.5%) were also used. Figure 5 summarizes the general psychological measures used in the studies.

### 4.3 Theme 3: evidence of emotional and behavioural symptoms in CV risk

Several CV risk factors have been investigated. Based on the deep neural network, the five CV risk factors most frequent in patients with T2DM were (1) BMI, (2) anxiety, (3) depression, (4) TC, and (5) systolic blood pressure (SBP). BMI is the strongest predictor of the incidence of CV complications (19). Shallcross et al. (20) highlighted that patients with treatment-resistant depression are more likely to be affected by diabetes and CVD (20). Pappaccogli et al. (21) demonstrated that managing stressful situations could emotionally impact patients, serving as the single predictor of BP control in those with resistant HTN (21).

Adherence to anti-hypertensive (AHT) drugs was significantly and positively correlated with all Health-Related Quality of Life (HRQOL) domains, with the exception of general health in hypertensive patients (22). An improvement in the QoL and self-efficacy was also reported in patients with T2DM (23) and, specifically, in the physical component in patients with HTN (24). In addition, the findings reported greater control of BP in hypertensive patients (24) and greater behavioural adherence by using app-based diet tracking plus feedback on DASH adherence via text message (25).

The common reasons for poor adherence among patients with HTN were forgetfulness, fear of side effects of medication, and financial cost. Poor adherence was more common in individuals aged <65 years and men. In addition, better exercise self-efficacy and adequate sleep (approximately 7 h per night) were positively associated with the likelihood of AHT adherence (26).

Personal beliefs are important in the management of disease. In fact, patients with T2DM who underwent an educational intervention based on sharing diabetes experiences showed a reduction in the fear of complications and improvements in BMI, HbA1c, SBP, and diastolic blood pressure (DBP) (27). Moreover, studies have confirmed the importance of mental disorders in the onset of significant health and wellbeing deficits. Finally, a positive correlation was identified among emotion dysregulation, distress, and self-care in patients with HTN (28).

With regard to cognitive processes, evidence has shown an association between improvement in cognitive domains and reduction in BMI, higher physical activity, and QoL in patients with MetS. In this study, higher adherence to the nutritional plan (Mediterranean Diet, MedDiet) was associated with improved memory, higher levels of executive functions, and global cognition, leading to better QoL; finally, women showed lower rates of change in global cognition, physical activity, and QoL (29). In addition, hypertensive women showed lower QoL and psychological health compared with hypertensive men (22).

### 4.4 Theme 4: evidence of emotional and behavioural symptoms in CV disease

A study showed that many factors contributed to the onset and maintenance of CV conditions, including the presence of apathy symptoms (30). Another study showed that patients with higher depressive symptoms had a 35% higher risk of recurrent fatal/non-fatal ACS incidence (31). These patients reported poorer sleep quality, higher trait anxiety, neuroticism, and negative affectivity. In addition, they had higher levels of social isolation, perceived stress, and functional impairment (32). One study showed that depressive symptoms were strongly associated with HRQOL (33). Patients with depressive symptoms were more likely to be non-adherent to their medication, engage in unhealthy behaviour (alcohol intake and smoking) (34), and report emotional distress more often (9). Another study showed that those with the highest MedDiet scores were less likely to experience elevated depressive symptoms (31). Emotional distress reported one year after an AMI was a weak predictor of statin adherence in the following year (9). Women with INOCA and distress more often had HTN, while men reported chest pain more often (35).

Depression plays a central role in worsening adherence to physical activity among patients with CV disease who do not receive a multidisciplinary intervention, and abnormal illness behaviour appears to deteriorate eating behaviour from 1 to 6 months after discharge (36). Therefore, the management of emotional aspects is crucial to achieve better health management. In fact, one study found that the change in depression score was also inversely and significantly associated with changes in the mental and physical component scores of QoL (33). These improvements were also observed in patients with HF (37). In addition, patients with CHF who underwent an integrated management intervention showed a reduction in depressive and anxious symptoms, better adherence, and better QoL. Mortality and hospital re-admissions for congestive HF were also reduced (38).

According to the classification of patients with CHF based on physical activity, high self-management and high tolerance to physical activity were identified only in group A, while groups B and C were not willing to engage in self-management activities (39).

A slight improvement in medical and behavioural adherence was observed after a secondary prevention intervention for the control of risk factors for ACS (40, 41). A well-recognized non-modifiable barrier to optimal secondary prevention is low socioeconomic status. It is also associated with poor cardiac prognosis (42).

Furthermore, the authors of a study reported that there was greater psychological wellbeing after cardiac rehabilitation (CR) in patients with VD. The authors also noted improvements in healthy behaviours, such as a better diet and greater physical activity (43).

Significant changes in protein intake and physical activity were observed from pre-event to the six-month follow-up in patients with ACS, suggesting the adoption of healthier behaviours. These improvements were observed mainly in patients with higher anxiety (44).

With regard to personality characteristics, an association was identified among Type D Personality, smoking, and poor adherence to statin therapy in patients with CAD. In particular, higher “negative affect” (NA) and lower “social inhibition” (SI) scores in outpatients with CAD were associated with an increased risk of recurrent CV events (45). Nevertheless, in another study, no association was identified among anxiety, depression, Type D personality, insomnia, perception of illness, and LDL-C control in patients with CV disease (42).

### 4.5 Theme 5: characteristics of patient adherence

**Figure 6:**
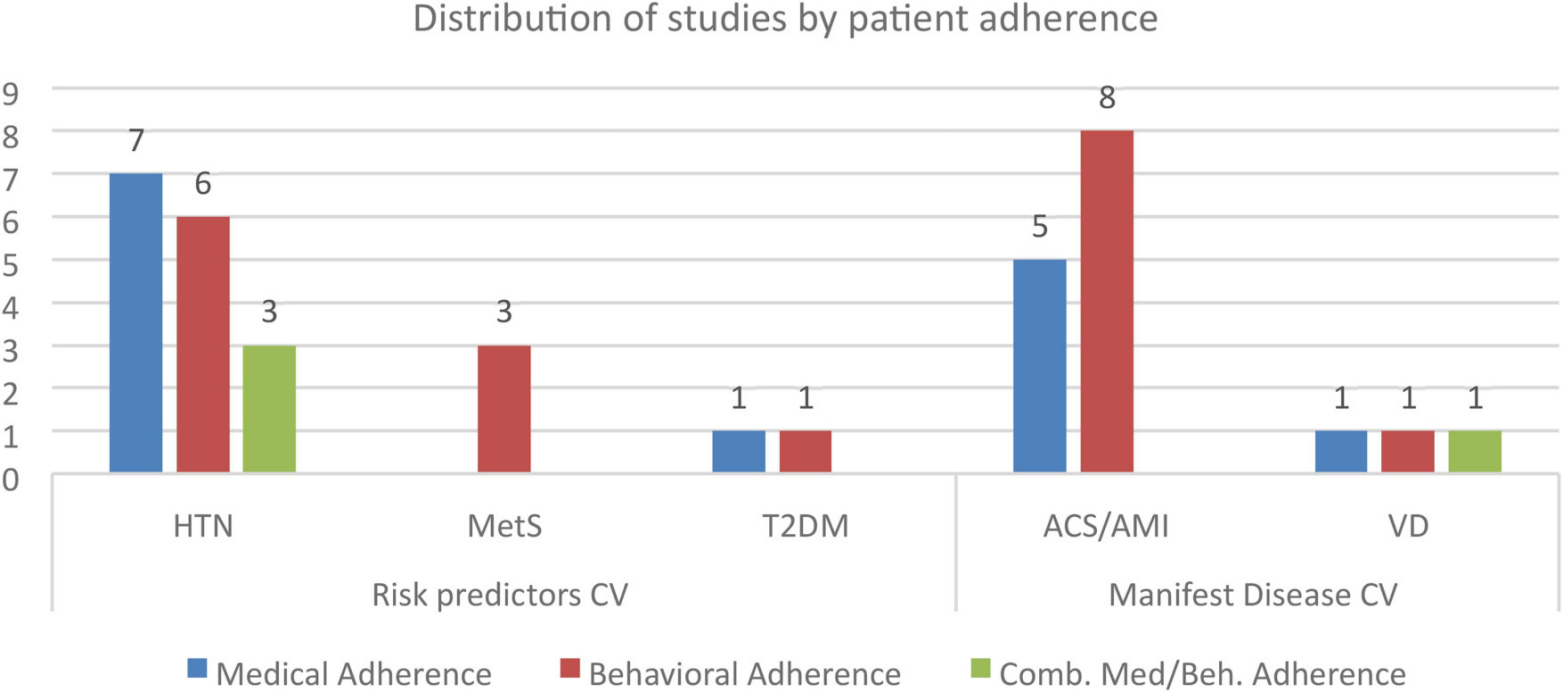
Distribution of studies by behavioural and medical adherence variables.

Figure 6 summarizes the patient adherence analysis in the examined studies. All research protocols focused on HTN and ACS/AMI detected behavioural and medical adherence data, even separately; few studies investigated combined behavioural and medical adherence (HTN: 3; VD: 1).

With regard to patient adherence, the included studies showed significant interest in behavioural adherence (36%), especially relative to dietary habits and physical activity. Adherence to medical treatment was studied based on both biomarker and behavioural components in 20% of the included studies. Methodologically, adherence to medical therapies was investigated using the Morisky Medication Adherence Scale (MMAS; *n* = 7, 18.4%) and the Medication Adherence Report Scale (MARS; *n* = 2, 5.3%).

With regard to psychological treatment, in numerous studies included in this review (71%), there was no reporting dedicated to the clinical study protocol, highlighting a lack of interest. The psychological interventions investigated in a few studies included the following: motivational interviewing (MI) (46, 47), cognitive behavioural therapy (CBT) (48, 49), chronic disease self-management program (CDSMP) (23), mindfulness-based blood pressure reduction (MB-BP) (50), ready to reduce risk (3R) (51), dietary approaches to stop hypertension (DASH) (25), systemic group education (group care) (27), health and wellness coaching (HWC) (52), positive psychology (PP), positive psychology motivational interviewing (PP-MI) (53), and psychosocial support (36, 38).

### 4.6 Theme 6: evidence of pre- and post-psychological treatment in CV risk/disease

As reported in Supplementary Table S1a, the efficacy of psychological interventions in the management of CV risk factors was studied. Patients with MetS reported significant improvements in WC, TG, and dietary behaviours at 3- and 6-month follow-ups after CBT (48). No differences in stress and QoL pre- and post-psychological intervention were identified (49). High behavioural adherence and biomarkers such as BMI, SBP, DBP, a reduction in anger, and CV risk calculated based on the Framingham score at 3-month post-treatment and 18-month follow-up were reported (49).

Other interventions also reported promising results: (a) 3R, (b) HWC, (c) MB-BP, (d) PP-MI, (e) multidisciplinary intervention, and (f) text messaging program based on the transtheoretical model.

Byrne et al. (51) studied the efficacy of the 3R psychoeducational intervention, contributing to the knowledge and awareness regarding a healthier lifestyle for reducing CV risk. Furthermore, this study introduced behavioural control techniques to support adherence to statins and a healthy lifestyle. Patients with high cholesterol levels undergoing 3R showed changes in WC and BP. They felt more in control of their treatment and had a more coherent understanding of their condition (51).

A recent study investigated the efficacy of an intervention based on PP principles. This HWC intervention was patient-centred and focused on health. Its goal was to increase the wellbeing of patients and improve the behaviours correlated to lifestyles. Patients with CV risk showed an improvement in BP control and a reduction in CV risk at 5 years compared with the control group at nine months. However, no differences in mood or QoL were identified (52).

Patients with HTN undergoing MI reported greater medical and behavioural adherence, better BP rates, and greater QoL and self-efficacy (47). In addition, they showed greater wellbeing, higher Hb1Ac levels, lower illness burden, and fewer depressive symptoms (46).

The MB-BP intervention was studied in patients with HTN. They reported an improvement in attention control, self-awareness, and emotional regulation from baseline to 1-year follow-up, as well as in aerobic and flexibility exercises. In addition, a reduction in SBP at 1-year follow-up and BMI at 3 months was observed (50).

The efficacy of psychological treatments in managing CVD is summarized in Supplementary Table S1b. Patients with ACS experienced an improvement in positive affect, depressive and anxious symptoms, and physical activity levels after PP-MI. These improvements were retained at the 12- and 24-month follow-ups (54). These results are consistent with the findings of other studies. In addition, Celano et al. (53) noted an increase in optimism, adherence, and self-reported physical activity post-intervention. These effects were observed even beyond 16 weeks (53).

Furthermore, patients with CV disease undergoing a multidisciplinary intervention showed improvements in stress management, quality of sleep, and dietary behaviour. Conversely, medication adherence appeared to be unaffected (36). This latter result was confirmed by a study of patients with ASCVD after a text message intervention involving behavioural modification techniques (55).

The limitations of the study are represented by the reduced examination of the statistical significance of the wellbeing outcome in patients with CV, as well as the preventive behaviour in CV risk; another limitation is the in-depth analysis of the interconnection between medical and behavioural dynamics in the evolution of cardiovascular risk/disease management from a lifelong perspective.

## 5 Implications for research and clinical practice

This review made a significant contribution to the field by highlighting the need to deal with patient factors in-depth according to the ESC Clinical Consensus Statement on mental health and CV disease (56) that addresses healthcare teams. Future investigations should address strategies to provide health management for patients with CV to be active actors, especially for patients who experience barriers to adapting to the disease.

This review demonstrates that interventions are required to address patient factors, such as emotional, behavioural, and cognitive dimensions, that facilitate better QoL and adherence to medication. The link between physical and mental care is important and should be supported by extensive evidence-based outcomes in terms of tailored actions in health monitoring and management of CV risk/disease clinical targets. The key point of the research protocols based on the BPS approach is the efficacy of integrated clinical treatments based on active patient engagement in efficient self-care.

## Data Availability

The original contributions presented in the study are included in the article/Supplementary Material, further inquiries can be directed to the corresponding author.
