# Supplementary material for: Biopsychosocial approach in cardiological care: a scoping review of integrated treatments for personalized patient care
Source: Front Cardiovasc Med. 2026 Mar 5;13:1727783. doi: 10.3389/fcvm.2026.1727783 (PMC13001114; doi:10.3389/fcvm.2026.1727783)
Supplement: Supplementary file 1 [file Datasheet1.pdf]

Table 1a. Main characteristics of the included studies with CV risk.

Study Design	Authors	Cardiovascular risk factors	Biomarkers/clinical data	Psychological measures	Psychological Intervention	Outcomes
RCT	Ma et al, 2014	HTN	SBP; DBP; Scr; TG; TC; LDLc; HDLc; FBG; PBG	TAQPH; GSES; SF-36	MI	ThTexte total scores and the mean scores for each dimension of the adherence questionnaire were increased in the intervention group, and the systolic blood pressure and diastolic blood pressure of the hypertensive patients greatly decreased in the intervention group during the six months of the motivational interviewing counselling
	Döbler et al, 2018	T2DM	HbA1c; BMI; BP	MARS; WHO-5; PHQ-9; PAID	MI	Patients in the intervention group showed improvement in psychological well-being, Hb1Ac, less illness burden and depressive symptoms.
	Garcia-Silva et al, 2018	MetS	BMI; WC; BP; Glycaemic; HDLc; TC; TG	MEDAS-14; FTND; MINI; MMSE	CBT	In interventions in which CBT was applied, significant improvements were observed in MetS patients, especially in adherence to the MedDiet, reduction in WC and TG at 3- and 6- months follow-up.
	Gamboa Moreno et al, 2019	T2DM	HbA1c; BMI; SBP; DBP	PAR; FFQ; ADDQoL-19; DSES-S	CDSMP	The intervention did not significantly modify HbA1c, or other cardiovascular variables. Significant improvements were seen in self-efficacy, and in particularly its disease control component. Certain differences were also observed in the use of healthcare resources and medication consumption.
	Loucks et al, 2019	HTN	BP, SBP, BMI	eCAPS; RAPA; FFQ; DASH Diet; BRFSS; MAIA; DERS; PSS-10; BAI; CESD-R; SART	MB-BP	The patients undergoing the intervention showed an improvement at one-year follow-up, including attention control, emotion regulation, and self-awareness. Several determinants of hypertension were improved in participants not adhering to American Heart Association guidelines at baseline, including physical activity, Dietary Approaches to Stop Hypertension-consistent diet, and alcohol consumption. Findings demonstrated reduction in SBP at one year follow-up.
	Byrne et al, 2020	HTN	TC; HDLc; DBP; SBP; BMI; WC	MMAS; IPAQ; FACET; EQ-5D; BMQ; B-IPQ	3R	No significant difference between the groups found for medication adherence to statins using either the urine test or other measures. This may have been due to the higher-than-expected adherence levels at baseline. The intervention group also showed greater perceived control of treatment and more coherent understanding of the condition.
	Steinberg et al, 2020	HTN	BMI; SBP; DBP	ASA-24; PHQ-8	DASH	A digital health intervention to improve DASH adherence is feasible and produces moderately high engagement among women with elevated blood pressure. The intervention did not enhance DASH adherence over diet tracking alone but resulted in greater reductions in blood pressure.
	Trento et al, 2020	T2DM	BMI; HbA1c; BP; Glycaemic; TC; TG; HDLc; LDLc; eGFR	HADS; DLC; DQOL; RSE	Group Care	Group Care patients showed an improvement on weight, BMI, HbA1c, SBP, DBP, dependence on Powerful Others in the Locus of Control Score. BP improvement among patients on Group Care was independent of BMI, duration of diabetes and antihypertensive medication, suggesting a direct effect of education, presumably by increasing adherence.
	Garcia-Silva et al, 2024	MetS	BMI; WC; SBP; DBP; HDLc; TC; TG; Glycaemic; Framingham Cardiovascular Risk Score	MEDAS-14; FTND; MINI; STAXI-2; AI; CACEV; PSS; SF-12	CBT	Results showed reduction in weight, waist circumference, body mass index, systolic and diastolic blood pressure, and cardiovascular risk score after 18 months. An increase in adherence to the Mediterranean diet and assertiveness and a reduction in anger were observed in EG. The CG did not show any significant differences.
	Krishnamurthi et al, 2024	HTN	BMI; fasting glucose; BP; TC	MMAS; EQ-5D; PHQ-9	HWC	HWC-group reported an improvement in control BP and a reduction of cardiovascular risk at 5-years. No statistically significant differences in mood scores, medication adherence, quality of life, and satisfaction with life scores over time or between groups were seen.
	Bolarinwa et al, 2019	HTN	BP; BMI	MMAS-8; SF-36	-	The between-group treatment effect was not statistically significant, whereas the within-group treatment effects were statistically significant for both the intervention and control arms at 6 months. After controlling for age and baseline HRQoL, the intervention group had an improved physical component of HRQoL than the control group. The intervention group also had statistically significant improvement in blood pressure control, medication adherence, and symptom counts.

	Soldevila-Domenech et al, 2021	MetS	BMI; BP; Glycaemic; HbA1c; HDLc; TC; TG	Er-MEDAS; RAPA; BDI-II; SF-36; RAVLT; RCFT; SDMT; Stroop; IGT; CPT; Vocabulary Test; MMSE	-	Baseline higher performance in verbal memory, visuoconstructive praxis and attention, and inhibition were associated with a higher odd of achieving at least 8% weight loss after 3 years follow-up in participants randomized to the intervention group. There were moderate improvements in specific tests of memory and executive functions during follow-up. Higher adherence to the er-MedDiet was associated with greater improvements in memory. Women exhibited lower rates of change in global cognition, PA and QoL. Moreover, improvements in memory correlated with reductions in BMI after 1 year and with improvements in PA after 3 years. Finally, participants who experienced greater improvements in executive functions and global cognition also experienced greater improvements in their QoL.
**OB**	Khalesi et al, 2017	HTN	BMI; SBP; DBP	FFQ; SF-12 V2	-	Three dietary patterns were identified from the FFQ, using factor and cluster analyses (Western, Snack and Alcohol, and Balanced). A Western dietary pattern, having lower exercise self-efficacy and shorter sleep duration were more dominant in the poor AHT adherence individuals compared to their counterparts. A positive association was observed between self-efficacy and sleep duration with AHT adherence. A Western dietary pattern was prevalent in high BP participants which slightly reduced the likelihood of good adherence.
	Shallcross et al, 2017	HTN	BP; HbA1c; BMI; Albuminuria; eGFR	KPAS; FFQ; GPSS; CESD-R; ISEL	-	Participants with apparent treatment-resistant hypertension had lower social network scores compared with participants without aTRH. Social network was independently associated with aTRH. No psychosocial factors differed between groups, except for regular social contact.
	Jneid et al, 2018	HTN	SBP; DBP	TSQM; WHOQOL-Brief	-	Better antihypertensive medication adherence was significantly and positively correlated with better HRQOL domains except general health where significance was not reached. Better adherence was also significantly related to better treatment satisfaction and an increased trust in physicians. Better treatment satisfaction (TSQM domains) was significantly and positively associated with a better overall HRQOL. Increased trust in physician scores were significantly and positively correlated with a better psychological health, environment, and overall HRQOL domains. Finally, TSQM effectiveness, convenience, and global satisfaction were significantly higher when trust in physician was greater
	Pappaccogli et al, 2019	HTN	BMI; BP	LC-MS/MS; TAS-20; ERQ; PTDS; BSI	-	At the end of follow-up only 26% of patients reached BP control. When compared to patients remaining resistant, patients eventually controlled had lower pulse pressure, less often myocardial infarction and showed a higher recourse to cognitive reappraisal as far as emotion regulation is concerned. Only the psychological characteristic of cognitive reappraisal (i.e., changing one's thoughts about a potentially emotion-eliciting event) remained significant.
	Chu et al, 2021	T2DM	HbA1c; Glycaemic; BMI; SBP; DBP; TC; HDLc; LDLc; TG	SAS; SDS	-	Among patients with T2DM, the top five predictors in the CVD risk model were body mass index, anxiety, depression, total cholesterol, and systolic blood pressure. In summary, machine learning models can provide an automated identification mechanism for patients at CVD risk. Integrated treatment measures should be taken in health management, including clinical care, mental health improvement, and health behavior promotion.
	Di Giacomo et al, 2024	HTN	SBP; DBP	DASS-21; DERS; SC-CII	-	Correlations among the main variables of interest showed a positive and significant relationship between emotional dysregulation indexes, psychological distress, and self-care domains (awareness resulted negatively and significantly correlated to self-efficacy); nonacceptance, goals and impulse indexes seemed positively and significantly correlated to anxiety and depression; finally, stress was correlated positively and significantly to awareness and impulse.

Legend:

Cardiovascular Risk Factors: HTN=Hypertension; T2DM=Type 2 Diabetes Mellitus; MetS=Metabolic Syndrome.

Biomarkers: SBP=Systolic Blood Pressure; DBP=Diastolic Blood Pressure; Scr=Serum creatinine; TG=Triglycerides; TC=Total cholesterol; LDLc=Low Density Lipoprotein-Cholesterol; HDLc=High-Density Lipoprotein-Cholesterol; FBG=Fasting Blood-Glucose; PBG=Postprandial Blood Glucose; Hb1Ac=Glycosylated Haemoglobin; BMI=Body Mass Index; BP=Blood Pressure; WC=Waist Circumference; eGFR=Estimated Glomerular Filtration Rate.

Psychological Measures: TAQPH=Treatment Adherence Questionnaire of Patients with Hypertension; GSES=General Self-Efficacy Scale; SF-36/12= Medical Outcomes Study 36/12-Item Short Form; MARS=Medication Adherence Report Scale; WHO-5=European Quality of Life Five Dimensions Questionnaire; PHQ-9/8=Patient Health Questionnaire-9/8; PAID=Problem Areas In Diabetes Scale; MEDAS-14=Mediterranean Diet Adherence Screener-14; FTND=The Fagerstrom Test for Nicotine Dependence; MINI=Mini International Neuropsychiatric Interview; MMSE=Mini Mental State Examination; PAR=Physical Activity Recall; FFQ=Food Frequency Questionnaire; ADDQoL-19=Audit of Diabetes-Dependent Quality of Life-19; DSES-S=Spanish Diabetes Self-efficacy Scale; eCAPS=Electronic Adherence Monitoring Device; RAPA=Rapid Assessment Physical Activity; DASH=Dietary Approaches to Stop Hypertension; BRFSS=Behavioral Factor Surveillance System Questionnaire; MAIA=Multidimensional Assessment of Interoceptive Awareness; DERS=Difficulties in Emotion Regulation Scale; PSS-10=Perceived Stress Scale-10; BAI=Beck Anxiety Inventory; CESD-R=Center for Epidemiology Study Depression Scale Revised; SART=Sustained Attention to Response Task; MMAS= Morisky Medication Adherence Scale; IPAQ=International Physical Activity Questionnaire; FACET=Five-A-Day Community Evaluation Tool; EQ-5D= European Quality of Life Five Dimensions Questionnaire; BMQ=Beliefs about Medicines Questionnaire; B-IPQ=Brief-Illness Perception Questionnaire; ASA-24=Automated Self-Administered 24-hour; HADS=Hospital Anxiety and Depression Scale; DLC=Diabetes Locus of Control; DQOL=Diabetes Quality Of Life; RSE=Rosenberg Self-Esteem Scale; STAXI-2=Inventory of Expression of Anger State-Trait; AI=Assertiveness Inventory; CACEV=Lifestyle-Related Assertiveness Questionnaire; PSS=Perceived Stress Scale; er-MEDAS=er-Mediterranean Diet Adherence Screener; BDI-II=Beck's Depression Inventory-II; RAVLT=Rey's Auditory-Verbal Learning Test; RCFT=Rey-Osterrieth Complex Figure Test; SDMT=Symbol Digit Modalities Test; IGT=Iowa Gambling Task; CPT=Conners' Continuous Auditory Test of Attention; SF-12 V2= Medical Outcomes Study 12-Item Version 2; KPAS=Kaiser Physical Activity Survey; GPSS=Global Perceived Stress Scale; ISEL=Interpersonal Support Evaluation List; TSQM=Treatment Satisfaction Questionnaire for Medication; WHOQOL-Brief=World Health Organization Quality of Life-Brief; LC-MS/MS=Liquid Chromatography-Mass Spectrometry; TAS-20=Toronto Alexithymia Scale-20; ERQ=Emotion Regulation Questionnaire; PTDS=Post Traumatic Diagnostic Scale; BSI=Brief Symptom Inventory; SAS=Self-Rating Anxiety Scale; SDS=Self-Rating Depression Scale; DASS-21=Depression Anxiety Stress Scale-21; SC-CII=Self-Care of Chronic Illness Inventory.

Psychological Treatment: MI=Motivational Interviewing; CBT=Cognitive Behavioural Therapy; CDSMP=Chronic Disease Self-Management Program; MB-BP=Mindfulness-Based Blood Pressure Reduction; 3R=Ready to Reduce Risk; DASH=Dietary Approaches to Stop Hypertension; Group Care=Systemic Group Education; HWC=Health and Wellness Coaching.

Table 1b. Main characteristics of the included studies with CV disease

Study Design	Authors	Cardiovascular condition	Biomarkers/clinical data	Psychological measures	Psychological Intervenitons	Outcomes
RCT	Villani et al, 2014	HF	SBP; DBP; HR; BMI; Potassium; Sodium; Glucose; Hb; Scr; BNP	MMAS; STAI-6; PHQ-9; PGWBI	Psychological Support	Integrated management patients showed better adherence, reduced anxiety and depression. In heart failure patients at high risk of relapse the possibility to contact the clinical staff improved psychological status and quality of life.
	Gostoli et al, 2016	CVD	BMI; LVEF	MMAS-8; PSQI; SCID; Interview based on DCPR; PFAMC	Psychosocial support	Cardiac rehabilitation was associated with maintenance of physical activity, improvement of behavioural aspects related to food consumption, stress management, and sleep quality. On the contrary, CR was not associated with weight loss, healthy diet, and medication adherence. Depression and psychosomatic syndromes seem to moderate the modification of specific health-related behaviours.
	Celano et al, 2018	ACS	BMI; LVEF	MOS-SAS; PANAS; LOT-R; HADS	PP- based intervention	The intervention was well accepted and associated with substantial improvements in behavioural and psychological outcomes. Booster sessions were associated with greater activity to a nearly significant degree, motivational interviewing was associated with overall adherence, and weekly exercise completion was generally superior to daily.
	Huffman et al, 2019	ACS	BMI	MOS-SAS; IPAQ; DASI-12; PANAS; HADS; SF-12; LOT-R	PP-MI	The PP-MI intervention was associated with large ES differences in improvement in positive affect as measured by the PANAS, the proximal target of the PP intervention component. The findings that the intervention had substantial effects on positive affect, depression, and anxiety, but not trait optimism, are consistent with a prior PP-alone intervention study in ACS patients. Authors also found that PP-MI was associated with greater physical activity.
	Bermon et al, 2021	ASCVD	LDLc; SBP; DBP; HR; Creatinine adjusted	MARS-5; PHQ-9	Text messaging program based on the Transtheoretical Model	There was no evidence that a behaviour modification intervention delivered by SMS text messaging improved LDL-C levels, blood pressure levels, or adherence at 12 months. More research is needed to evaluate whether different SMS text messaging strategies, including personalized messages and different timings, are effective; future studies should include mixed methods to better understand why, for whom, and in which context (eg, health system or social environment) SMS text messaging interventions work (or not) to improve adherence in patients with ASCVD.
	Marcos-Forniol et al, 2018	ACS	SBP; DBP; BMI; LDLc; HbA1c; CCI	MET; FFQ; OARS-IADL; SPPB; Barthel Index; CCI SF-36	-	After the 12-month intervention, 34.2% more patients in the intervention group had achieved optimum risk factor control compared with usual care. Blood pressure, LDL-c, HbA1c and physical activity were better controlled in the intervention group than in usual care.

	Yudi et al, 2021	ACS	HbA1c; SBP; DBP; BMI; WC; TC; HDLc; LDLc; TG; eGFR	6MWT; CDS; DS-SF; HADS; SF-36; EQ-5D	-	At 8-week follow-up, the S-CRP group had a clinically significant improvement in 6-minute walk test distance. Patients in the S-CRP were more likely to participate and adhere to a cardiac rehabilitation program. Compared to UC, patients receiving S-CRP had similar smoking cessation rates, LDLcholesterol levels, blood pressure reduction, depression, anxiety and quality of life measures.
OB	Vollmer-Conna et al, 2015	ACS	BMI; CRP; IL-1β; IL-6; TNF-α; IL-10, IFN-γ; 25[OH]D; vitamin D, cytokines and peak troponin T; HR	MINI; PSQI; DMI-10; PSQ; DS-14; BDQ; EPQ-R; CCAS; DFSS	-	The majority of participants with ACS-associated and ongoing depression were members of the class characterised by the greatest biological disturbance. Patients with depression differed from those without depression on a range of psychological trait and state variables; additionally reporting poorer sleep quality, higher levels of social isolation, and functional impairment, but had similar biological profiles. Patients with ongoing depression generally had higher scores on these psychological/behavioural measures. This study identified a combination of biomarkers suggestive of a role for immune, autonomic, and nutritional pathways in the manifestation of depression during ACS, in the context of additional psychosocial and behavioural vulnerabilities.
	Munkhaugen et al, 2017	AMI; angina	TC; HDLc; LDLc; BMI; BP; CRP; CCI	MMAS; HADS; PSWQ; BIS	-	Statin specific side-effects, low statin adherence and moderate- or low-intensity statin therapy were the major factors associated with unfavourable LDL-C control. Interventions to improve LDL-C should ensure adherence and prescription of sufficiently potent statins, and address side-effects appropriately. No association between anxiety, depression, insomnia, and illness perception, respectively, and LDL-C control.
	Berg et al, 2018	Risk/Disease CV	BMI	HADS	-	Depressive patients had 34% higher odds of being non-adherent to their medication. At one-year follow-up, patients with depression had the highest attributable risk associated with mortality followed by: smoking, ischemic heart disease, anxiety, diabetes, hypertension chronic obstructive pulmonary disease and excessive alcohol consumption. Depression and anxiety in patients with cardiac disease is associated with cardiac risk behaviour such as smoking, obesity, excessive alcohol consumption and medication non-adherence.
	Doi et al, 2020	HF	BNP; LVEF; SBP; DBP; BMI	SAS; EHFScBS; SF-8	-	There were significant differences in the New York Heart Association (NYHA) class, BNP level, self-management of medication adherence and exercise habits.
	Mommersteeg et al, 2021	CAD	BMI	BDI; HADS	-	Consistent distress was more prevalent in women and was related to the use of more angiotensin-converting enzyme inhibitors/angiotensin receptor blockers and diuretics in women and to calcium antagonist use as well as lower adherence levels in men. Women who reported chest pain more often received angina relief medication and blood-pressure-lowering medication than men. No sex differences were observed in cardiac medication use in patients with suspected INOCA. Psychological distress may reflect hypertension and subsequent medication use in women, and experiencing chest pain and subsequent medication use in men.
	Eurelings et al, 2014	CVD	BMI; SBP; TC	ALDS; GDS-15; MMSE	-	Apathy, but not depression, is a strong, independent risk factor for incident CVD. Apathy was associated with incident CVD after adjustment for demographics and cardiovascular risk factors. No association was found between depressive symptoms and incident CVD. Neither apathy symptoms nor depressive symptoms were associated with incident stroke

Kowal et al, 2015	CD; VD	BMI; HDLc; LDLc; WC; SBP; SDP; HbA1c	MMAS-4; DASI-12; GLTEQ; HPLP-II; CRBS; PHQ-8; EQ-5D	-	Cardiac patients who attended CR achieved significant improvements in activity status, exercise behaviour, and nutrition at the post-test. Among VD patients, there were trends toward lower depressive symptoms and greater exercise in those who participated in CR by post-test.
Notara et al, 2016	ACS	CK; CK-MB; Troponin I; ECG; BMI	FFQ; MedDietScore; CES-D	-	ACS coexisting with severe depression status seems to result in adverse disease outcomes while financial status and Mediterranean diet are proposed as potential moderators. Patients with depressive symptoms have a higher risk of 35% of incidence of fatal/recurrent not fatal ACS.
Weiss et al, 2019	HF; LVD; ICM	LVEF; HR; SBP; DBP; BMI; GFR; TSH; NT-pro-BNP	SF-12; BDI-II; STAI	-	At enrolment 21% showed clinically significant depressive symptoms and 52% anxiety symptoms. At 6 weeks, depressive and anxiety symptoms significantly decreased. Depressive symptoms at enrolment and changes at 6 weeks showed significant association with health-related quality of life, whereas anxious symptoms did not.
Torgersen et al, 2023	CAD	LDLc; CRP; SBP; WC	HADS; DS-14	-	Higher negative affectivity (NA) score was associated with Major adverse cardiovascular events (MACE) after adjustment for age, risk factors and comorbidity Low statin adherence and smoking were more prevalent in the Type D personality and NA group. The NA trait is related to worse prognosis in outpatients with CAD.
Lissåker et al, 2017	AMI	BMI; TC; LVEF	MPR; EQ-5D; MINI; BAI; BDI	-	Emotional distress two months after the MI was not associated with statin adherence one year later but emotional distress reported one year after the MI was a weak predictor of statin adherence one year later.
Heo et al, 2018	HF	LVEF; sodium; SSQ-HF; CCI	MLHFQ; PHQ-9; CAS-R; MSPSS	-	Improvement in HF symptoms were associated with improvement in physical and emotional domain of quality of life over 12 months. Changes in HF symptoms were significantly associated with the likelihood of at least a 5-point improvement in quality of life.
Greco et al, 2021	ACS	BMI; WC; SBP; DBP	MDS; RAPA-Q; HADS	-	Changes in protein intake and physical activity were found from pre-event to the six-month follow-up. soon after the six-month follow-up, patients experienced significant declines in their healthy behaviors. Both physical activity and red/processed meat intake were modulated by the season in which the assessments took place and by anxiety symptoms over time.

Legend:

Cardiovascular Disease: HF=Heart Failure; CVD=Cardiovascular Disease; ACS=Acute Coronary Syndrome; ASCVD=Atherosclerotic Cardiovascular Disease; AMI=Acute Myocardial Infarction; CV=Cardiovascular; CAD=Coronary Artery Disease; CD=Cardiac Disease; VD=Vascular disease; LVD=Left-Ventricular Dysfunction; ICM=Ischemic Cardiomyopathy.

Biomarkers: SBP=Systolic Blood Pressure; DBP=Diastolic Blood Pressure; HR=Heart Rate; BMI=Body Mass Index; Hb=Hemoglobin; Scr=Serum Creatinine; BNP=Brain Natriuretic Peptide; LVEF=Left Ventricular Ejection Fraction; LDLc=Low Density Lipoprotein-Cholesterol; Hb1Ac=Glycosylated Haemoglobin; CCI=Charlson Comorbidity Index; WC=Waist Circumference; TC=Total Cholesterol; HDLc=High-Density Lipoprotein-Cholesterol; TG=Triglycerides; eGFR=Estimated Glomerular Filtration Rate; CRP=C-Reactive Protein; IL-1β=Interleukin-1β; IL-6=Interleukin-6; TNF-α=Tumor Necrosis factor-α; IL-10=Interleukin-10; IFN-γ=Interferon-γ; 25[OH]D=Serum 25-hydroxyvitamin D; BP=Blood Pressure; CK=Creatine Kinase; CK-MB=MB Fraction of Total CK; ECG=Electrocardiogram; GFR=Glomerular Filtration Rate; TSH=Thyroid Stimulating Hormone; NT-pro-BNP=N-Terminal-pro-brain Natriuretic Peptide; SSQ-HF= Symptom Status Questionnaire-Heart Failure.

Psychological Measures: MMAS-8/4= Morisky Medication Adherence Scale-8/4; STAI-6=Spielberger's State Trait Anxiety Inventory-6; PHQ-9/8=Patient Health Questionnaire-9/8; PGWBI=Perceived General Well Being Index; PSQI=Pittsburgh Sleep Quality Index; SCID=Structured Clinical Interview for DSM-IV-TR; Interview based on the DCPR= Interview based on the Diagnostic Criteria for Psychosomatic Research; PFAMC=Psychological Factors Affecting Medical Condition; MOS-SAS=Medical Outcomes Study Specific Adherence Scale; PANAS=Positive and Negative Affect Schedule; LOT-R=Life Orientation Test-Revised; HADS=Hospital Anxiety and Depression Scale; IPAQ=International Physical Activity Questionnaire; DASI-12=Duke Activity Status Index-12; SF-12/36/8= Medical Outcomes Study 12/36/8-Item Short Form; MARS-5=Medication Adherence Report Scale-5; MET=Metabolic Equivalent of Task; FFQ= Food Frequency Questionnaire; OARS-IADL=Older Americans' Resources and Services Activities of Daily Living; SPPB=Short Physical Performance Battery; CCI=Charlson Comorbidity Index; 6MWT=6-Minute Walk Test; CDS=Cardiac Depression Scale; DS-SF=Depression Scale-Short Form; EQ-5D= European Quality of Life Five Dimensions Questionnaire; MINI=International Neuropsychiatric Interview; DMI-10=Depression in the Medically Ill-short version; PSQ=Perceived Stress Questionnaire; DS-14=Type D Personality Assessment-14; BDQ=Brief Disability Questionnaire; EPQ-R=Eysenck Personality Questionnaire-Revised; CCAS=Costello and Comrey Anxiety Scale; DFSS=Duke-UNC Functional Social Support Questionnaire; PSWQ=Penn State Worry Questionnaire; BIS=Bergen Insomnia Scale; SAS=Specific Activity Scale; EHFScBS=European Heart Failure Self-Care Behaviour Scale; BDI/II=Beck Depression Inventory/II; ALDS=Academic Medical Center Linear Disability Score; GDS-15=15-item Geriatric Depression Scale; MMSE=Mini Mental State Examination; GLTEQ=Godin Leisure-Time Exercise Questionnaire; HPLP-II=Health Promoting Lifestyle Profile II; CRBS=Cardiac Rehabilitation Barriers Scale; CES-D=Center for Epidemiological Studies-Depressive symptoms; STAI=State Trait Anxiety Inventory; MPR=Medication Possession Ratio; BAI=Beck Anxiety Inventory; MLHFQ=Minnesota Living Heart Failure questionnaire; CAS-R=Control Attitudes Scale-Revised; MSPSS=Multidimensional Scale of Perceived Social Support; MDS=Mediterranean Diet Scale; RAPA-Q=Rapid Assessment of Physical Activity Questionnaire.

Psychological Treatment: CR=Cardiac Rehabilitation; PP=Positive Psychology; PP-MI=Positive Psychology-Motivational Interviewing.
